# Transient simulation of laser ablation based on Monte Carlo light transport with dynamic optical properties model

**DOI:** 10.1038/s41598-023-39026-4

**Published:** 2023-07-24

**Authors:** Yu Shimojo, Kazuma Sudo, Takahiro Nishimura, Toshiyuki Ozawa, Daisuke Tsuruta, Kunio Awazu

**Affiliations:** 1 Graduate School of Medicine, Osaka Metropolitan University, Asahimachi 1-4-3, Abeno-ku, Osaka, 545-8585 Japan; 2 Graduate School of Engineering, Osaka University, Yamadaoka 2-1, Suita, Osaka 565-0871 Japan; 3 Research Fellow of Japan Society for the Promotion of Science, Kojimachi 5-3-1, Chiyoda-ku, Tokyo, 102-0083 Japan; 4 Global Center for Medical Engineering and Informatics, Yamadaoka 2-2, Suita, Osaka 565-0871 Japan

**Keywords:** Biomedical engineering, Biophotonics

## Abstract

Laser ablation is a minimally invasive therapeutic technique to denature tumors through coagulation and/or vaporization. Computational simulations of laser ablation can evaluate treatment outcomes quantitatively and provide numerical indices to determine treatment conditions, thus accelerating the technique’s clinical application. These simulations involve calculations of light transport, thermal diffusion, and the extent of thermal damage. The optical properties of tissue, which govern light transport through the tissue, vary during heating, and this affects the treatment outcomes. Nevertheless, the optical properties in conventional simulations of coagulation and vaporization remain constant. Here, we propose a laser ablation simulation based on Monte Carlo light transport with a dynamic optical properties (DOP) model. The proposed simulation is validated by performing optical properties measurements and laser irradiation experiments on porcine liver tissue. The DOP model showed the replicability of the changes in tissue optical properties during heating. Furthermore, the proposed simulation estimated coagulation areas that were comparable to experimental results at low-power irradiation settings and provided more than 2.5 times higher accuracy when calculating coagulation and vaporization areas than simulations using static optical properties at high-power irradiation settings. Our results demonstrate the proposed simulation’s applicability to coagulation and vaporization region calculations in tissue for retrospectively evaluating the treatment effects of laser ablation.

## Introduction

Laser ablation is an electromagnetically-based thermal technique that provides minimally invasive treatment for benign lesions and malignant tumors^1^. Absorption of the laser energy by the tissue induces coagulation and vaporization through photothermal conversion. These photothermal effects are able to denature and remove lesions while concurrently inhibiting bleeding through coagulation, thereby reducing the risk of adverse events while also maintaining a blood-free surgical field^2,3^. Therefore, laser ablation has been clinically applied to various tissue types, including liver^4,5^, prostate^6,7^, brain^8^, and cervix tissues^9^. The efficacy and safety of the treatment outcomes are dependent not only on the optical properties of the tissue but also on the irradiation parameters used, which comprise the irradiation wavelength, the irradiation power, and the irradiation duration. The effects of these factors have been evaluated using animal models and tissue-mimicking phantoms^10,11^. Animal models are heterogeneous across specimens, resulting in the requirement for a large number of samples to accurately evaluate the effects of laser ablation^12^. Phantoms are homogeneous and may provide more robust models to investigate the photothermal responses^13^. Nevertheless, because of the qualitative nature of the models, determination of the appropriate treatment conditions from the enormous number of possible combinations of the irradiation parameters remains difficult. A model for quantitative evaluation of the relationships between the treatment outcomes and the treatment conditions is therefore required.

To evaluate laser ablation quantitatively, numerous studies have been conducted with the aim of developing mathematical models of tissue photothermal responses^14–16^. These models enable computational simulations to quantify the photothermal effects of laser ablation. Computational simulations of laser ablation consist of calculations of the light transport, the temperature increase, and the degree of thermal damage caused^17–19^. Each step requires an input of numerical information from the previous step, experimental measurement of the tissue properties, and modeling of the tissue structure. The temperature increase causes changes in the optical properties of the tissue, affecting the reduced scattering $\mu _\mathrm{{s}}'$ and absorption $\mu _\mathrm{{a}}$ coefficients, and these changes then affect the light transport in the tissue^20^. The heat-induced changes in the tissue optical properties can be characterized using the Arrhenius integral^21^. Previous studies have shown that a dynamic optical properties (DOP) model using the Arrhenius integral can predict the tissue coagulation induced by a relatively moderate temperature increase^22,23^. The light transport calculations in these studies used the diffusion approximation with a diffusing optical fiber model. Similar to this method, a Monte Carlo (MC) method can also model the light transport in tissue with the incorporation of a diffusing optical fiber^24^. The MC model can produce the light distribution in the tissue with complex geometries and inhomogeneities^25^, and can more accurately estimate the light distribution in the tissue near the light source where the coagulation and vaporization processes occur than the diffusion approximation^26,27^. So far, laser vaporization simulations using MC models of light transport in tissue have been proposed^18,19^. The vaporization process is modeled by taking a phase change into consideration. However, the optical properties during heating have been assumed to remain constant during the process. The effects of heating on the optical properties of the tissue should be evaluated in situations in which the tissue undergoes a phase change because the changes in the optical properties of the tissue will increase with increasing temperature^28^.

This study proposes a novel computational simulation approach to laser ablation based on MC light transport with a DOP model. To validate the DOP model, the optical properties of porcine liver tissue are measured experimentally as a function of the ratio of thermally-damaged tissue $\alpha$. The optical properties simulated using the DOP model are then incorporated into the proposed simulation to calculate the light absorption distribution within the tissue. Subsequently, the thermal diffusion and thermal damage are calculated based on the light absorption distribution to determine the vaporization and coagulation regions. The simulation results are compared with the results that were obtained using static optical properties, i.e., the simulation results without use of the DOP model, and are validated by performing ex vivo experiments using porcine liver tissue and a laser diode operating at a wavelength of 980 nm. The proposed simulation allows us to increase the accuracy of vaporization and coagulation regions estimation for evaluating the treatment effects after the procedure of laser ablation.

## Computational simulation of laser ablation

**Figure 1:**
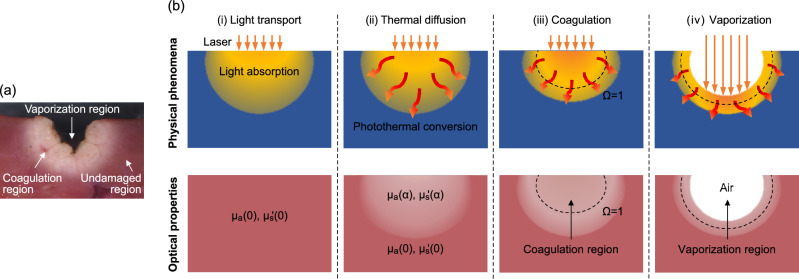
(**a**) Cross-section of laser-irradiated tissue. (**b**) Schematic illustrations of the photothermal responses and the corresponding changes in the tissue’s optical properties and structure during laser irradiation. The changes in the tissue’s optical properties and structure affect the light distribution in the tissue. The broken lines indicate the point at which $\Omega =1$, i.e., where $\alpha =63\%$. $\Omega$: damage parameter; $\alpha$: ratio of thermally-damaged tissue; $\mu _\mathrm{{s}}'(\alpha )$: reduced scattering coefficient; and $\mu _\mathrm{{a}}(\alpha )$: absorption coefficient.

During laser ablation, tissue undergoes coagulation and/or vaporization upon exposure to the laser light, as depicted in Fig. 1a. At the tissue surface, vaporization resulted in tissue removal to form a crater shape, where the ablated tissue transitions into the air. The tissue around the vaporization region has turned white because of coagulation, which indicates that a change in the optical properties from their native state has occurred during laser irradiation. Figure 1b shows a schematic illustration of the photothermal responses and the corresponding changes in the tissue’s optical properties and structure during heating by laser irradiation. First, the irradiated laser light is absorbed by the tissue (i). The absorbed laser energy then generates heat through photothermal conversion and, at the same time, the heat diffuses into the tissue (ii). This thermal diffusion causes the temperature to rise and increases the ratio of thermally-damaged tissue $\alpha$. This photothermal response causes continuous changes in the optical properties of the tissue, i.e., the coefficients $\mu _\mathrm{{s}}'(\alpha )$ and $\mu _\mathrm{{a}}(\alpha )$. Coagulation is known to occur when the tissue temperature reaches approximately 60 °C because of protein denaturation. However, the extent of the thermal damage is dependent not only on the temperature but also on the tissue’s exposure time at that temperature^17^. The ratio $\alpha$ is thus quantified using the temperature- and time-dependent damage parameter $\Omega$, which is calculated using the Arrhenius integral (see Eq. 1 below)^29^. When $\Omega =1$, i.e., when $\alpha =63\%$ (the threshold for irreversible damage; see Eq. 2), the tissue is then assumed to have coagulated (iii)^29^. Tissue vaporization occurs when the tissue temperature reaches and is maintained at its boiling point (iv). This process is assumed to be a water vaporization process^18^. When the enthalpy of the tissue exceeds a specific threshold, the tissue is then removed and the optical properties and structure of the tissue change to those of air. In the simulations, high-power irradiation settings (comprising an irradiation power of 50 W and movement speeds of 0.5, 0.75, and 1.0 mm/s) and low-power irradiation settings (comprising an irradiation power of 10 W and movement speeds of 1.0, 1.25, and 1.5 mm/s) are implemented with the intention of inducing both coagulation and vaporization and of inducing coagulation alone, respectively. The values for the movement speeds were chosen based on the values available in clinical practice^30^.

## Theory of change in tissue optical properties during heating

The Arrhenius integral is used to evaluate the relationships between the optical properties $\mu _\mathrm{{s}}'(\alpha )$ and $\mu _\mathrm{{a}}(\alpha )$ and the thermal damage ratio of the tissue $\alpha$^29^. This is a kinetic model of irreversible thermal alteration to the tissue and calculates a nondimensional damage parameter $\Omega (r,t)$ (r: position vector in tissue and t: time), which is dependent on both the temperature and the exposure time, from:

1 $$\begin{aligned} \Omega (\textbf{r},t) = \ln \left\{ \frac{C(\textbf{r},0)}{C(\textbf{r},t)} \right\} = A \displaystyle \int _0^t \exp {\left\{ \frac{E_\mathrm{{a}}}{R T(\textbf{r},t)} \right\} } \mathrm{{d}}t, \end{aligned}$$

where *C* is the remaining concentration of undamaged tissue, *A* (1/s) is a frequency factor, $E_{\text{a}}$ (J/mol) is the activation energy, *R* (J/(mol$\cdot$K)) is the gas constant, and *T* is the temperature. The ratio $\alpha$ can be calculated using the following equation:

2 $$\begin{aligned} \alpha (\textbf{r},t) = 1-\exp \{-\Omega (\textbf{r},t)\}. \end{aligned}$$

During light transport through the tissue at near-infrared wavelengths, scattering is a more dominant phenomenon than absorption. In addition, previous studies have reported that there is almost no variation in $\mu _\mathrm{{a}}(\alpha )$ during heating when compared with $\mu _\mathrm{{s}}'(\alpha )$^31^. Therefore, the changes in the tissue optical properties are modeled based on the assumption that the change in $\mu _\mathrm{{a}}(\alpha )$ due to thermal damage can be considered to be negligible. From Mie theory, $\mu _\mathrm{{s}}'(\alpha )$ can be calculated based on the volume fractions of scatters. Therefore, the DOP model can be written as follows:

3 $$\begin{aligned} \mu _\mathrm{{s}}'(\alpha ) = (1-\alpha (\textbf{r},t))\mu _\mathrm{{s,native}}'+\alpha (\textbf{r},t) \mu _\mathrm{{s,coagulated}}', \end{aligned}$$

where $\mu _\mathrm{{s,native}}'$ (mm$^{-1}$) is $\mu _\mathrm{{s}}'(\alpha )$ for the native tissue and $\mu _\mathrm{{s,coagulated}}'$ (mm$^{-1}$) is $\mu _\mathrm{{s}}'(\alpha )$ for the completely coagulated tissue. The optical properties simulated using the DOP model are then input into the computational simulation to calculate the tissue coagulation and vaporization.

## Results

### Optical properties of the native and coagulated tissues

**Figure 2:**
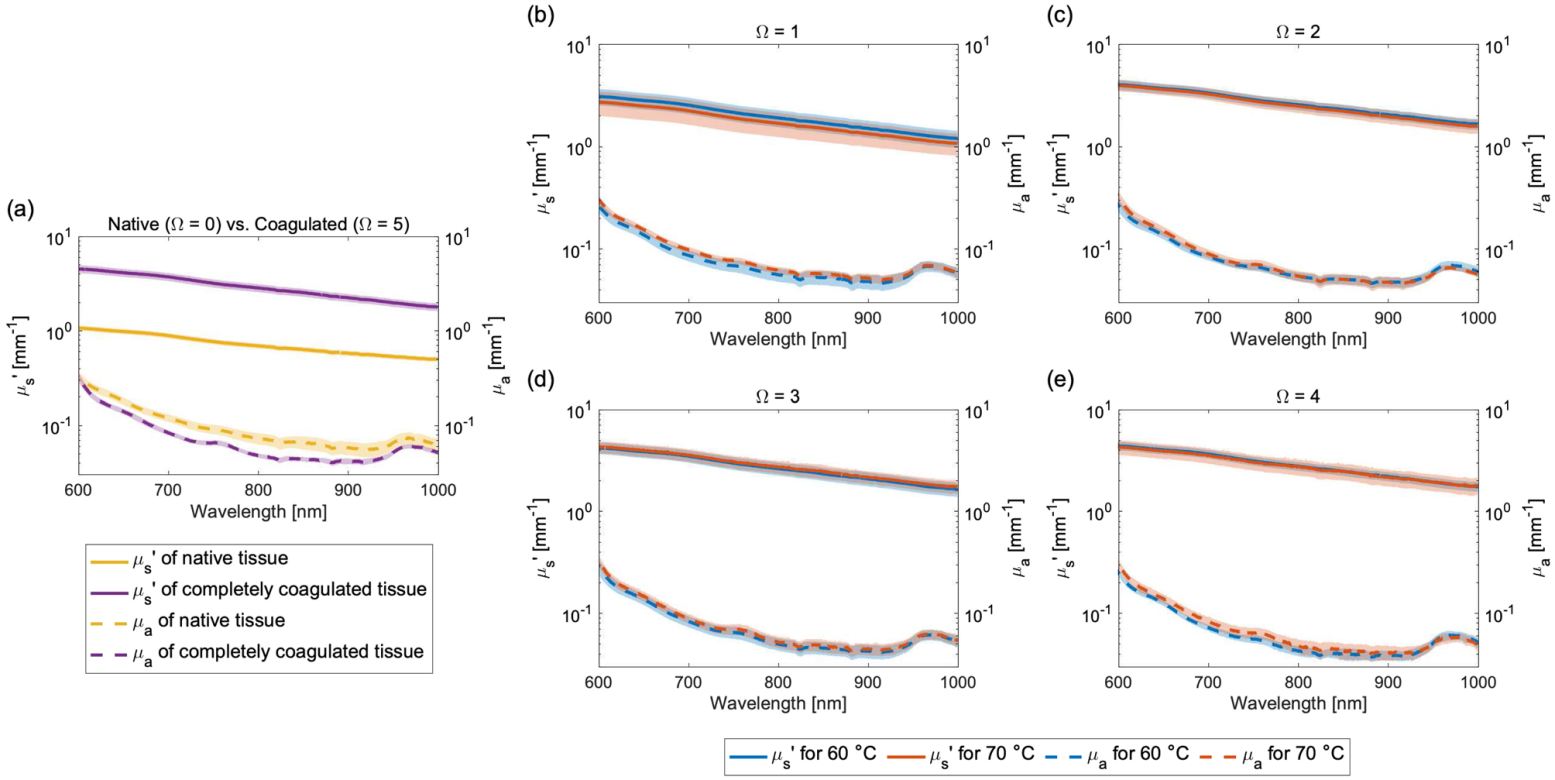
Reduced scattering and absorption coefficient spectra of porcine liver tissue. (**a**) Comparison between spectra of native tissue ($\Omega =0$) and completely coagulated tissue ($\Omega =5$). (**b**–**e**) Comparison between spectra under bathing temperature conditions of 60 and 70 °C for $\Omega =1, 2, 3,$ and 4. The shaded areas represent the standard deviation in each case.

Figure 2a shows the $\mu _{\text{s}}^{\prime}$ and $\mu _{\text{a}}$ spectra of both the native porcine liver tissue and the completely coagulated tissue, which was bathed at 70 °C ($\Omega =5$, i.e., $\alpha =99\%$), within the 600–1100 nm wavelength range. The value of $\mu _{\text{s}}^{\prime}$ decreased monotonically with increasing wavelength, while the value of $\mu _{\text{a}}$ showed a water absorption peak at approximately 980 nm. The $\mu _{\text{a}}$ value decreased by a factor of at most 0.36 because of coagulation, whereas the $\mu _{\text{s}}^{\prime}$ value increased by a factor of at least 2.52 within the wavelength range under study. Therefore, $\mu _{\text{s}}^{\prime}$ was found to be more sensitive to coagulation-induced changes than $\mu _{\text{a}}$. Furthermore, Fig. 2b–e show the $\mu _{\text{s}}^{\prime}$ and $\mu _{\text{a}}$ spectra of the thermally-damaged tissues, which were bathed at temperatures of 60 and 70 °C for different $\Omega$ values. Interestingly, almost no differences were observed between the $\mu _{\text{s}}^{\prime}$ and $\mu _{\text{a}}$ spectra at 60 and 70 °C for each $\Omega$.

### Validation of the DOP model

**Figure 3:**
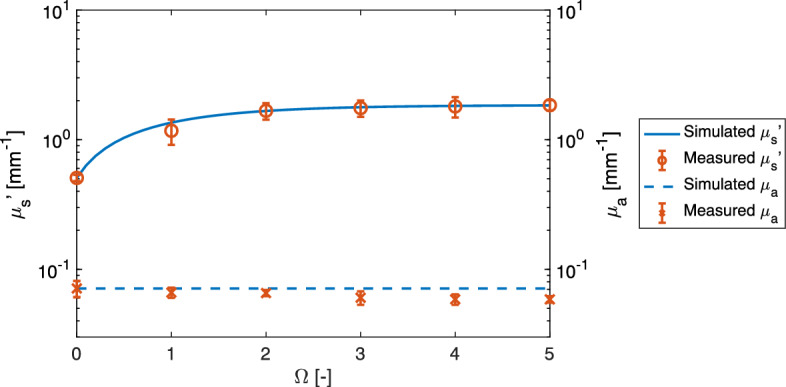
Comparison between simulated and measured reduced scattering and absorption coefficients at a wavelength of 980 nm for different damage parameter values. The error bars represent the standard deviation.

Figure 3 shows the optical properties of the tissue at 980 nm after being bathed at 70 °C versus $\Omega$ and the corresponding optical properties of the tissue when simulated using the DOP model. The root mean square percentage error (RMSPE) between the simulated and measured $\mu _{\text{s}}^{\prime}$ values was 9% at 980 nm. The simulated $\mu _{\text{s}}^{\prime}$ characteristic agreed well with the measured $\mu _{\text{s}}^{\prime}$ values. This agreement was observed across all the wavelengths investigated (see Supplementary Fig. S1). Both the simulated and measured $\mu _{\text{s}}^{\prime}$ values increased with increasing $\Omega$, and the rate of this increase was highest up to $\Omega =1$, where $\mu _{\text{s}}^{\prime}$ increased by a factor of 1.31 when compared with that of the native tissue. From $\Omega =1$ to 2, $\mu _{\text{s}}^{\prime}$ increased by a factor of 0.43, and the rate at which the coefficient increased diminished as $\Omega$ increased. This suggests that $\mu _{\text{s}}^{\prime}$ increases rapidly up to $\Omega =1$, which represents the threshold for irreversible reaction, and then increases only moderately beyond that threshold. These findings indicate that the DOP model can replicate the shift in $\mu _{\text{s}}^{\prime}$ during heating successfully. The measured $\mu _{\text{a}}$ values were $0.07\pm 0.01$ mm$^{-1}$ at $\Omega =0$ and $0.06\pm 0.01$ mm$^{-1}$ at $\Omega =5$. At the wavelength of 980 nm, the influence of tissue coagulation on $\mu_{\text{a}}$ was negligible.

### Vaporization and coagulation for the high-power irradiation settings

**Figure 4:**
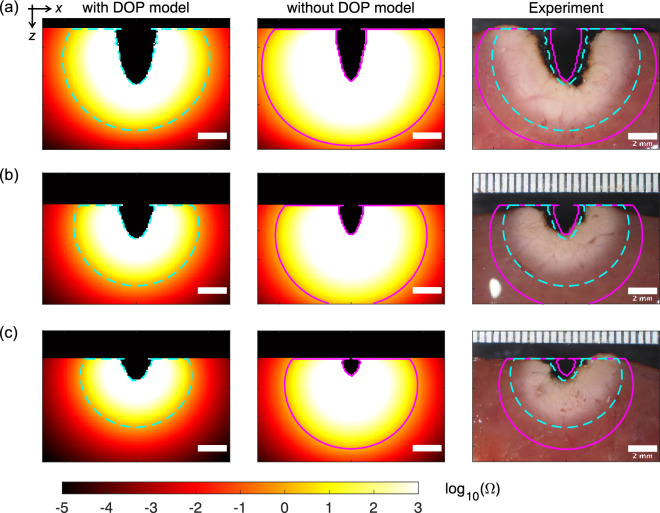
Thermal damage distributions on the *zx* plane with $y=0$ for an irradiation power of 50 W and movement speeds of (**a**) 0.5, (**b**) 0.75, and (**c**) 1.0 mm/s from the simulations performed with and without the DOP model and from the experiments (Supplementary Movie S1). The cyan broken lines and the magenta solid lines show the tissue regions where $\Omega$ is equal to 1. The laser beam diameter was 2.8 mm. Scale bar: 2 mm.

**Figure 5:**
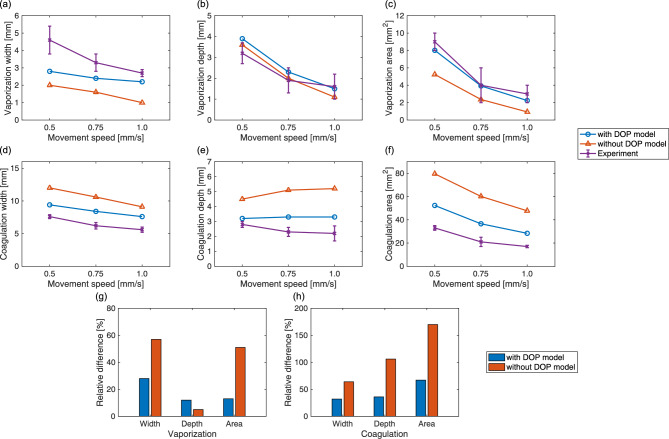
Quantitative comparison of the vaporization and coagulation regions obtained from the simulations with and without the DOP model and those obtained experimentally for the high-power irradiation settings. (**a**) Widths, (**b**) depths, and (**c**) areas of the vaporization regions, and (**d**) widths, (**e**) depths, and (**f**) areas of the coagulation regions. Three independent experiments were performed for each irradiation condition. The error bars represent the standard deviation. Absolute values of relative differences in (**g**) vaporization width, depth, and area, and (**h**) coagulation width, depth, and area between the simulated and experimental results obtained with and without the DOP model.

Figure 4 shows the extent of the thermal damage that resulted from use of the high-power irradiation settings from both the simulations and the experiments. Supplementary Movie S1 shows the spatiotemporal changes in the simulated thermal damage distributions on the *zx* plane. Vaporization and coagulation were both observed near the tissue surface. The shapes of the vaporization regions simulated using the DOP model in the *z* direction were sharper when compared with those observed in the experiments. The coagulation regions simulated using the DOP model were larger than those from the experimental results. When compared with the simulation results obtained without the DOP model, the vaporization regions simulated using the DOP model were larger, while the coagulation regions simulated using the DOP model were smaller, resulting in better agreement with the experimental results. Figure 5 presents the quantitative details of the vaporization widths, depths, and areas, and the coagulation widths, depths, and areas. In both the simulations and the experiments, the vaporization widths, depths, and areas, and the coagulation widths and areas all decreased with increasing movement speed. In contrast, the coagulation depths remained nearly constant at the different movement speeds in both the simulation using the DOP model and the experiments. However, the coagulation depth increased with increasing movement speed for the simulation performed without the DOP model. The relative differences between the simulated and experimental results obtained with and without the DOP model when averaged by movement speed were 28 and 57% for the vaporization widths; 12 and 5% for the vaporization depths; 13 and 51% for the vaporization areas; 32 and 64% for the coagulation widths; 36 and 106% for the coagulation depths; and 67 and 170% for the coagulation areas, respectively. The vaporization and coagulation areas simulated using the DOP model showed differences from the experimental results that were approximately four times and 2.5 times smaller, respectively, when compared with the simulation results obtained without the DOP model.

### Coagulation for the low-power irradiation settings

**Figure 6:**
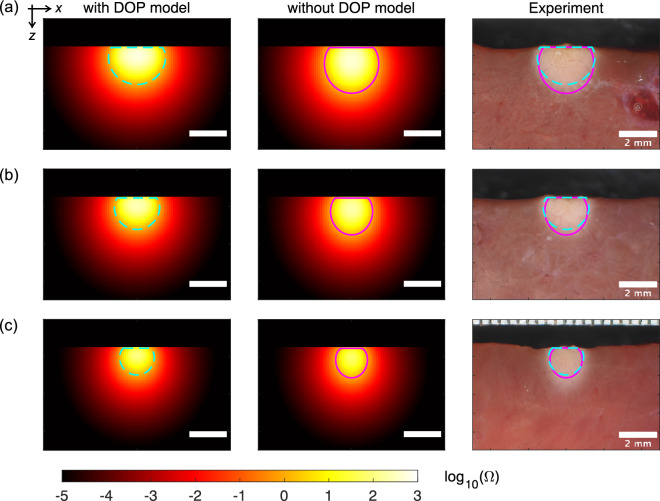
Thermal damage distributions on the *zx* plane with $y=0$ for an irradiation power of 10 W and movement speeds of (**a**) 1.0, (**b**) 1.25, and (**c**) 1.5 mm/s from the simulations with and without the DOP model and the experiments (Supplementary Movie S2). The cyan broken lines and the magenta solid lines indicate the tissue regions where $\Omega$ is equal to 1. The laser beam diameter was 2.8 mm. Scale bar: 2 mm.

**Figure 7:**
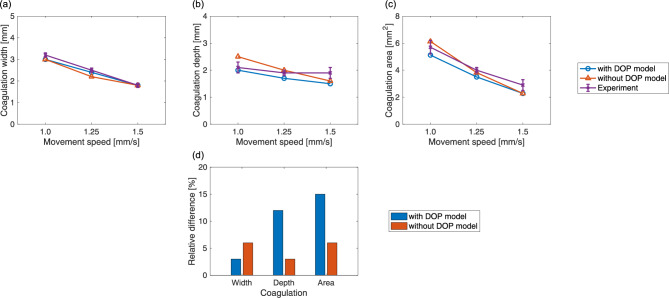
Quantitative comparisons of the coagulation regions between the simulations with and without the DOP model and the experiments for the low-power irradiation settings. (**a**) Widths, (**b**) depths, and (**c**) areas. Three independent experiments were performed for each irradiation condition. The error bars represent the standard deviation. (**d**) Absolute values of relative differences in coagulation width, depth, and area between the simulated and experimental results obtained with and without the DOP model.

Figure 6 shows the extent of the thermal damage that resulted from use of the low-power irradiation settings from both the simulations and the experiments. Supplementary Movie S2 shows the spatiotemporal changes in the simulated thermal damage distributions that occurred on the *zx* plane. Notably, coagulation occurred near the tissue surface but vaporization did not occur. Both thermal damage distributions, when simulated with and without the DOP model, were comparable to the experimental distribution. However, the thermal damage distributions obtained with the DOP model showed a wider distribution in the *x* direction and a narrower distribution in the *z* direction when compared with those obtained without the DOP model. Quantitative comparisons of the coagulation widths, depths, and areas are shown in Fig. 7. The coagulation widths, depths, and areas all decreased linearly with increasing movement speed for both the simulations and the experiment. The relative differences between the simulated and experimental results with and without the DOP model when averaged by movement speed were 3 and 6% for the coagulation widths; 12 and 3% for the coagulation depths; and 15 and 6% for the coagulation areas, respectively. Therefore, the simulation results with and without the DOP model both demonstrated almost no difference from the experimental results.

### Effect of the DOP model on vaporization and coagulation regions estimation

**Figure 8:**
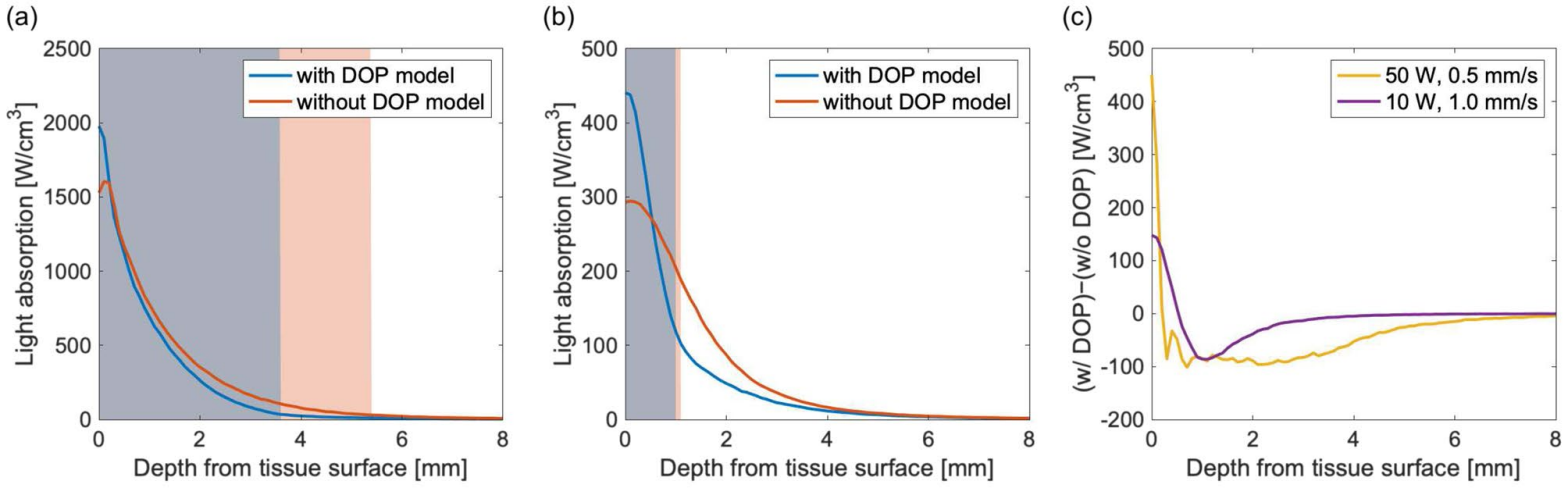
Light absorption distributions from the tissue surface on the *z* axis when the laser light was incident at $x=y=0$ mm, calculated with and without the DOP model for (**a**) an irradiation power of 50 W and a movement speed of 0.5 mm/s, and (**b**) an irradiation power of 10 W and a movement speed of 1.0 mm/s. The blue and orange shaded areas represent the coagulation region obtained with and without the DOP model, respectively. (**c**) Differences between the light absorption distributions calculated with and without the DOP model.

Figure 8a shows the light absorption distributions obtained from the surface of the vaporized tissue for the high-power irradiation setting, while Fig. 8b shows the light absorption distributions from the tissue surface for the low-power irradiation setting, when the laser light was incident at $x=y=0$ mm. The yellow and purple lines in Fig. 8c show the difference between the results obtained with and without the DOP model for the high- and low-power irradiation settings, respectively. Because the simulation with the DOP model took the increase in $\mu _{\text{s}}^{\prime}$ into account, the light diffusion into the tissue was lower than that in the simulation performed without the DOP model. Therefore, the coagulation regions obtained via the simulation with the DOP model were smaller than those obtained from simulations without the DOP model. For the high-power irradiation settings, the reduced light diffusion resulted in greater light absorption near the tissue surface, which increased vaporization. This caused a greater degree of vaporization to occur in the simulation performed with the DOP model than in the simulation performed without the DOP model. These findings showed that the proposed simulation can predict the vaporization and coagulation characteristics of tissue when caused by a severe temperature increase that causes the tissue to undergo a phase change. For the low-power irradiation settings, the simulated amount of light absorption without the DOP model was greater than that determined by the DOP model in the deeper region below the coagulation region (i.e., at depths greater than 1 mm). However, this difference was less than half of that observed in the high-power irradiation settings case. Therefore, the proposed simulation had little effect on the coagulation region when using the low-power irradiation settings.

## Discussion

This study proposed a 3D transient simulation of laser ablation based on MC light transport with a DOP model. The changes in the tissue optical properties were modeled by considering the volume fractions of the thermally-damaged tissue and the remaining undamaged tissue, using the Arrhenius integral. The $\mu _{\text{s}}^{\prime}$ values simulated using the DOP model demonstrated consistency with the $\mu _{\text{s}}^{\prime}$ values measured for different values of $\Omega$. The MC and enthalpy methods were used to calculate the light transport and the subsequent thermal diffusion in the tissue, respectively, reflecting the changes in the optical properties and structure of the tissue caused by thermal damage. The simulated vaporization and coagulation regions were compared with the corresponding simulation results obtained without the DOP model and were validated quantitatively through ex vivo experiments. The results of the comparison and the validation showed improved accuracy in estimation of the vaporization and coagulation regions. Use of this simulation allows for quantification of the effects of laser ablation with a simple operation of setting irradiation parameters on a computer. Consequently, treatment effects can be compared across various combinations of irradiation parameters to evaluate the appropriate treatment conditions. Furthermore, using monitoring modalities that use the change in optical properties to quantify ablation region^32,33^, the ablation region obtained from irradiation under the evaluated treatment conditions can be evaluated. Therefore, the presented simulation in combination with these modalities has the potential to improve the efficacy and safety of laser ablation.

In the DOP model, the change in $\mu _{\text{a}}$ during heating was disregarded because the scattering is more dominant than the absorption during light transport in tissue at the wavelength of 980 nm. The optical properties measurements indicated that $\mu _{\text{s}}^{\prime}$ at 980 nm of the samples changed by a factor of 2.65 because of coagulation, while $\mu _{\text{a}}$ at 980 nm changed by only a factor of 0.18 (see Fig. 2a). Consequently, this study focused on $\mu _{\text{s}}^{\prime}$ when modeling the changes in tissue optical properties during heating. However, the measured $\mu _{\text{a}}$ slightly decreased with increasing $\Omega$. This change results in an 8% increase in optical penetration depth^25^, estimated by $\delta =1/\sqrt{3\mu _\mathrm{{a}}(\mu _\mathrm{{a}}+\mu _\mathrm{{s,coagulated}}')}$, and at least 18% decrease in light absorption near the tissue surface, potentially leading to a reduction in vaporization and coagulation regions. The assumption regarding $\mu _{\text{a}}$ may result in an overestimation of vaporization and coagulation regions.

Although the change in optical properties was modeled at the near-infrared wavelength, further verification is required to apply the same model to other wavelength ranges. A previous study found a decrease in the hemoglobin absorption peak at 415 nm because of coagulation^20^. Additionally, water absorption peaks in tissue at mid-infrared wavelengths shift because of high water temperatures. Adapting the DOP model for use at the visible and mid-infrared wavelengths could pose theoretical challenges. Therefore, considering modeling of the change in $\mu _{\text{a}}$ during heating will be essential for wavelengths at which absorption by chromophores, such as hemoglobin and water, is comparable to or more dominant than scattering by tissue to expand the range of wavelengths where the proposed simulation is applicable.

In the thermal diffusion model, the temperature dependences of the thermal properties were neglected. A previous study on microwave thermal ablation reported that the temperature dependences of the thermal properties had little effect on the coagulation area at temperature below 60 °C, while at temperature above 90 °C, considering the temperature dependences of the thermal properties yielded coagulation areas differed by 10–16% compared to the case where the temperature dependences were ignored^34^. The assumption regarding thermal properties had little impact on the simulation results at the low-power irradiation settings, but may introduce differences at the high-power settings. In the thermal damage model, the coagulation region was determined based on the assumption of $\Omega =1$. Although this assumption is common in laser ablation simulations, the Arrhenius parameters, i.e., *A* and $E_{\text{a}}$, exhibit variations depending on individuals, tissue types, and tissue temperatures^29^. This parameter uncertainty may affect the simulation results.

Discrepancies were observed in the vaporization width and the coagulation width, depth, and area between the simulation results with the DOP model and the experimental results. Theses discrepancies likely arose from the fact that the DOP model did not consider carbonization of the tissue. Carbonization increases light absorption at the tissue surface and also reduces light penetration into the tissue, which would then increase the vaporization width and reduce the degree of coagulation^35^. Integration of a mathematical carbonization model into the proposed simulation will improve the accuracy of vaporization and coagulation regions estimation^36^. Another factor may be the difference in sample preparation between the optical properties measurements and the laser irradiation experiments. The simulation results were obtained using the simulated optical properties, which mimicked the experimentally measured values. Notably, the samples used for the optical properties measurements were in direct contact with water for bathing, whereas the samples used for the laser irradiation experiments were not. The absorption of water can alter the optical properties of tissues. Furthermore, the coagulation regions in the laser irradiation experiments were determined based on the dataset obtained from samples coagulated in water (see Supplementary Fig. S6). These assumptions in experimental conditions may contribute to the differences between the simulation and experimental results of vaporization and coagulation regions. For the low-power irradiation settings with and without the DOP model (see Figs. 6 and 7), almost no differences were observed in the coagulation region between the simulations and the experimental results. Previous studies have reported successful coagulation estimation when using the DOP model in a laser ablation simulation^37^, while others have realized successful estimates by considering the static optical properties^17^. These previous findings support the results obtained via our simulations.

The improved estimation accuracy facilitates the determination of appropriate irradiation parameters for laser ablation. The simulation results obtained without the DOP model underestimated the vaporization regions when compared with the simulations performed with the DOP model (see Fig. 5c). This underestimation indicates that the simulation performed without the DOP model could provide parameters that lead to over-irradiation, which could cause thermal damage to the normal tissue around the targeted diseased tissue. Use of the proposed simulation in clinical practice may lead to improved safety for laser ablation in terms of the associated thermal damage. Note, however, that both our simulations and the ex vivo experiments neglected the cooling effect of blood perfusion. Moreover, the presented simulations used the optical properties of the frozen-thawed ex vivo tissues, which are different from in vivo tissue optical properties^38^. They did not also consider clinical variables including intra- and inter-patient variability in optical and thermal properties. These factors limit the clinical applicability of the presented simulation. Measurement techniques for these parameters in vivo have been proposed^39^. In conjunction with such techniques, the proposed simulation will achieve more accurate estimation of vaporization and coagulation regions, contributing to the improvement of the efficacy and safety of laser ablation.

Although a collimated laser beam was used for both the simulations and the ex vivo experiments, the laser light is generally guided by an optical fiber for clinical treatment of deeply seated lesions and of lesions in locations that are difficult to access^40^. Various probe shapes are available, depending on the target tissue and the laser ablation technique used^41,42^. MC simulations can incorporate different diffusing optical fibers and beam shapes to calculate light distribution in tissue^24,43^. Development of light source models will allow application of the proposed simulation to both treatment planning systems and computational clinical trials for laser ablation.

In conclusion, this study presents a laser ablation simulation based on MC light transport with a DOP model. Optical properties measurements confirmed that the DOP model can reproduce the changes that occur in the tissue’s optical properties during heating. Comparison of the simulation results with the ex vivo experimental results showed that the proposed simulation can calculate the vaporization and coagulation areas with more than 2.5 times higher accuracy in the high-power irradiation settings case when compared with the conventional simulation. Future work will involve integration of a mathematical model of carbonization into the proposed simulation to enhance the accuracy when predicting the vaporization and coagulation regions of the tissue.

## Methods

### Flowchart of laser ablation simulation

The flowchart for the computational simulation of the laser ablation process is shown in Supplementary Fig. S2. First, a numerical tissue model $M(\textbf{r},t)$ containing information about the tissue structure and the optical and thermal properties of the tissue was constructed. Second, an MC simulation of the light transport in the tissue was performed to calculate the light absorption distribution $S(\textbf{r},t)$. It was assumed that the absorbed light was converted instantaneously into heat. Third, the spatiotemporal distributions of the enthalpy $H(\textbf{r},t)$ and the temperature $T(\textbf{r},t)$ were calculated by solving the governing equation using the finite difference method. Subsequently, the damage parameter $\Omega (\textbf{r},t)$ was quantified based on the temperature distribution using the Arrhenius integral. The tissue was regarded as being coagulated when $\Omega$ exceeded 1, and when $H(\textbf{r},t)$ reached a threshold enthalpy, the tissue was removed and changed into the air. Finally, the tissue model $M(\textbf{r},t)$ was reconstructed based on the changes in the tissue’s properties and structure. During the calculation procedure, the calculation time required for the thermal diffusion and the thermal damage $t_{\text{heat}}$ when the value of $\Omega$ hardly changed during light transport was considered to reduce the total calculation time $t_{\text{total}}$. The iteration count for the thermal diffusion and thermal damage calculations was defined as $i_{\text{heat}}$. The calculation continued while the value of $i_{\text{heat}}\times \Delta t$ was less than $t_{\text{heat}}$. The iteration count for the light transport calculation was then defined as $j_{\text{light}}$. The light absorption distribution was then updated unless $j_{\text{light}}\times t_{\text{heat}}$ exceeded the laser irradiation time $t_{\text{laser},{\text {on}}}$. Immediately after laser irradiation, the tissue was still so hot that the remaining heat could cause the tissue to coagulate. The thermal diffusion and thermal damage calculations continued for the post-laser irradiation time $t_{\text{laser},{\text {off}}}$ until the tissue temperature decreased. The total calculation time $t_{\text{total}}$ was given by the sum of $t_{\text{laser},{\text {on}}}$ and $t_{\text{laser},{\text {off}}}$. The simulation codes were implemented using C and Compute Unified Device Architecture (CUDA, Nvidia).

### Numerical model of tissue

A 3D numerical model of the tissue consisting of air and tissue layers was constructed using cubic voxels composed of $300\times 300\times 150$ elements. The voxel dimensions were $0.1~\textrm{mm} \times 0.1~\textrm{mm} \times 0.1~\textrm{mm}$. The depth direction was set to be along the *z* axis. The tissue surface was set at *z* = 5 mm. The plane vertical to the *z* axis was defined as the *xy* plane. The tissue surface center was located at $(x,y)=(0~\textrm{mm},0~\textrm{mm})$. The thicknesses of the air and the tissue were set at 5 and 10 mm, respectively. The optical and thermal properties used when performing the computational simulations are summarized in Supplementary Table S1. Because scattering is more dominant than absorption within the near-infrared wavelength range during light transport through the tissue, the change in $\mu _{\text{a}}$ during heating was assumed to be negligible. The $\mu _{\text{s}}^{\prime}$ value varied according to Eq. 3. The refractive index of the tissue was maintained at 1.44 during heating in the calculations because of the limited nature of the available experimental data^21^. The thermal properties were assumed to remain invariant during heating for simplicity in this study. Future work should integrate the temperature dependences of the thermal properties into the proposed simulation to improve the computational accuracy of the laser ablation^44^.

### MC simulation of light transport in tissue

An MC simulation called Monte Carlo eXtreme (MCX) was used to calculate the light absorption distribution in the tissue at a wavelength of 980 nm^45^. This computational simulation enables calculation of the light absorption distribution in complex tissue structures composed of multiple types of tissue, each with its own optical properties. The simulations used a collimated laser beam similar to that used in the experiments (see Supplementary Fig. S5), as the objective of this study was to evaluate the impact of the DOP model on vaporization and coagulation regions through a comparison of the simulation and experimental results. The laser beam irradiated the tissue surface vertically and moved from $(x,y)=(0~\textrm{mm},-10~\textrm{mm})$ to $(0~\textrm{mm},10~\textrm{mm})$. The beam profile had a Gaussian distribution and the beam diameter (1/$e^2$) was 2.8 mm. We set the high-power irradiation settings (an irradiation power of 50 W and movement speeds of 0.5, 0.75, and 1.0 mm/s) with the intention of inducing both coagulation and vaporization, and set the low-power irradiation settings (an irradiation power of 10 W and movement speeds of 1.0, 1.25, and 1.5 mm/s) with the intention of inducing only coagulation. The simulations were performed for photon packets of $1.0\times 10^7$ photons to achieve sufficient light absorption distributions in the tissue.

### Thermal diffusion and thermal damage simulation

Laser tissue coagulation was simulated by applying $\Omega =1$ as the threshold for irreversible damage^29^. Laser tissue vaporization was assumed to be a water vaporization process^18^. The pressure was assumed to remain constant under atmospheric pressure conditions^14^. The temperature rise induced by the laser irradiation was calculated using the enthalpy method. The governing equation is shown as Eq. 4:

4 $$\begin{aligned} \frac{\partial H(\textbf{r},t)}{\partial t} = k \nabla ^2 T(\textbf{r},t) + S(\textbf{r},t), \end{aligned}$$

where *k* [W/(cm$\cdot$K)] is the thermal conductivity. This equation was solved using the finite difference method. The adiabatic condition was applied to the boundaries. The temperature was related to the enthalpy as follows:

5 $$\begin{aligned} T({r},t)= \left\{ \begin{aligned}& \frac{H({r},t)}{\rho c_{\text{p}}} \quad (H<\rho c_{\text{p}}T_{\text{v}})\\& T_{\text{v}}\quad (\rho c_{\text{p}}T_{\text{v}}<H<\rho (c_{\text{p}}T_{\text{v}}+L_{\text{v}}))\\ \end{aligned} \right. , \end{aligned}$$

where $\rho$ (g/cm$^{3}$) is the density, $c_{\text{p}}$ (J/(g$\cdot$K)) is the specific heat capacity, $T_{\text{v}}$ (°C) is the vaporization temperature, and $L_{\text{v}}$ (J/g) is the latent heat of vaporization. The initial temperature for both the air and the tissue was set at 22 °C to simulate room temperature. $\Omega$ was then calculated from the temperature distribution obtained using the Arrhenius integral (see Eq. 1). The enthalpy distribution obtained was compared with a threshold enthalpy that was defined as:

6 $$\begin{aligned} H_{\text{th}}=\rho (c_{\text{p}}T_{\text{v}}+L_{\text{v}}). \end{aligned}$$

When *H* exceeded $H_{\text{th}}$, the tissue was removed and changed into the air. For the simulation conditions, $\Delta t$ was set at $1.0\times 10^{-5}$ s, $t_{\text{heat}}$ was set at $2.0\times 10^{-3}$ s, and $t_{\text{laser}},{\text {off}}$ was set at 30 and 10 s for the high-power irradiation settings and the low-power irradiation settings, respectively. $\Delta t$ satisfied the stability criterion when solving Eq. 4.

### Measurement of the tissue optical properties

The optical properties of the tissue samples were measured using a double integrating sphere spectrophotometer and the inverse MC technique, as described previously^46^. Porcine liver tissue samples (purchased from Tokyo Shibaura Zouki) were used as an alternative to human liver tissue. The samples were frozen and then thawed in order to slice them into the dimensions of approximately $15~\textrm{mm} \times 15~\textrm{mm} \times 1.5~\textrm{mm}$. The thickness of 1.5 mm was confirmed using a micrometer. To prepare samples with a variety of damage parameters, the liver tissue was coagulated using a hot water bath. The tissue was in direct contact with water. Tissue coagulation typically occurs when the temperature exceeds 60 °C^47^. Therefore, the bathing temperature was set at 60 and 70 °C to evaluate the temperature dependence of the change in the tissue’s optical properties. Bathing time was determined from numerical simulations, which are described in Supplementary Figs. S3 and S4 and Supplementary Table S2. Each coagulated sample was then cooled using distilled water at 4 °C to prevent additional thermal damage. The sample thickness was measured again using a micrometer to evaluate the shrinkage caused by heating, but almost no changes were observed. The sample was sandwiched between glass slides and the sample thickness was then fixed using spacers. The sample was sealed with adhesive tape to prevent desiccation from occurring during the measurements. Ten samples were prepared for each bathing condition. All prepared samples were stored at room temperature (22 °C) before the measurements were taken. In the inverse analysis, the anisotropy factor *g* was assumed to be 0.9, which is a typical value in many tissues^48^. The refractive index *n* was also assumed to be 1.44.

### Laser irradiation experiment

To validate the simulation results, ex vivo experiments were conducted using a 980 nm laser and porcine liver tissue (see Supplementary Fig. S5). A laser diode (980LD-6-4-1, Aerodiode) was used to cause coagulation and vaporization of the samples. A pulsed laser source was not used in our experiments because a high power density of a pulsed laser can induce nonlinear absorption by biological tissue, thereby complicating the modeling of light transport in tissue^49^. Instead, a collimated laser beam was used to compare the simulation results with the experimental results. Three samples were prepared for each irradiation condition and were stored at room temperature (22 °C) prior to irradiation. The laser beam emitted from the multimode fiber (core diameter of 106 $\mu$m and numerical aperture of 0.22) was collimated using an aspheric lens (ACL2018U, Thorlabs) and reflected from a mirror (PF10-03-P01, Thorlabs). The reflected beam was then directed toward the sample. The spatial distribution of the laser beam at the sample surface was measured using a complementary metal-oxide-semiconductor (CMOS) camera (resolution of 5 $\mu$m/pixel). The beam profile had a Gaussian distribution with a beam diameter (1/$e^2$) of 2.8 mm. The profile and diameter for the collimated laser beam were consistent with those used in the MC simulations of light transport in tissue (see Supplementary Fig. S5(c)). The sample to be irradiated was placed on a motorized linear translation stage and scanned for 2 cm. The irradiation power was measured using a power sensor. The irradiated samples were stored at $-20$ °C after the experiments and were then cut at the center of their scanned range to enable observation of the sample cross-sections with a color camera attached to an optical microscope. To enable quantitative comparison of the extent of the thermal damage, the vaporization depths, widths, and areas, and the coagulation depths, widths, and areas were measured using ImageJ (see Supplementary Fig. S6)^17^.

## Supplementary Information

Supplementary Information 1.

Supplementary Information 2.

Supplementary Information 3.

## Data Availability

The datasets used and/or analyzed during the current study are available from the corresponding author on reasonable request.

## References

[1] Kok HP (2020). Heating technology for malignant tumors: A review. Int. J. Hyperth..

[2] Nair SM, Pimentel MA, Gilling PJ (2016). A review of laser treatment for symptomatic BPH (benign prostatic hyperplasia). Curr. Urol. Rep..

[3] Katta N, Estrada AD, McElroy AB, Milner TE (2022). Er:YAG laser brain surgery with vascular specific coagulation. Lasers Surg. Med..

[4] Sartori S, Di Vece F, Ermili F, Tombesi P (2017). Laser ablation of liver tumors: An ancillary technique, or an alternative to radiofrequency and microwave?. World J. Radiol..

[5] Tombesi P, Vece FD, Sartori S (2015). Radiofrequency, microwave, and laser ablation of liver tumors: Time to move toward a tailored ablation technique?. Hepatoma Res..

[6] Miyazaki, H. *et al.* Early experiences of contact laser vaporization of the prostate using the 980 nm high power diode laser for benign prostatic hyperplasia. *LUTS: Lower Urin. Tract Symp.* **10**, 242–246 (2018). 10.1111/luts.12173 28573791

[7] Shaker, H., Alokda, A. & Mahmoud, H. The Twister laser fiber degradation and tissue ablation capability during 980-nm high-power diode laser ablation of the prostate. A randomized study versus the standard side-firing fiber. *Lasers Med. Sci.* **27**, 959–963 (2012). 10.1007/s10103-011-1017-8 PMC3414700 22071987

[8] Medvid R (2015). Current applications of MRI-guided laser interstitial thermal therapy in the treatment of brain neoplasms and epilepsy: a radiologic and neurosurgical overview. Am. J. Neuroradiol..

[9] Randall TC, Sauvaget C, Muwonge R, Trimble EL, Jeronimo J (2019). Worthy of further consideration: An updated meta-analysis to address the feasibility, acceptability, safety and efficacy of thermal ablation in the treatment of cervical cancer precursor lesions. Prev. Med..

[10] Stafford RJ (2010). Magnetic resonance guided, focal laser induced interstitial thermal therapy in a canine prostate model. J. Urol..

[11] Van Nimwegen S, L’eplattenier H, Rem A, Van Der Lugt J, Kirpensteijn J (2008). Nd:YAG surgical laser effects in canine prostate tissue: Temperature and damage distribution. Physics in Medicine & Biology.

[12] Geoghegan R (2019). A tissue-mimicking prostate phantom for 980 nm laser interstitial thermal therapy. Int. J. Hyperth..

[13] Negussie AH (2016). Thermochromic tissue-mimicking phantom for optimisation of thermal tumour ablation. Int. J. Hyperth..

[14] Blauth S, Hübner F, Leithäuser C, Siedow N, Vogl TJ (2020). Mathematical modeling of vaporization during laser-induced thermotherapy in liver tissue. J. Math. Ind..

[15] Shimojo Y, Nishimura T, Hazama H, Ito N, Awazu K (2020). Picosecond laser-induced photothermal skin damage evaluation by computational clinical trial. Laser Therapy.

[16] Shimojo Y, Nishimura T, Hazama H, Ito N, Awazu K (2021). Incident fluence analysis for 755-nm picosecond laser treatment of pigmented skin lesions based on threshold fluences for melanosome disruption. Lasers Surg. Med..

[17] Tran VN, Truong VG, Jeong S, Kang HW (2018). Computational analysis of linear energy modulation for laser thermal coagulation. Biomed. Opt. Express.

[18] Elkhalil H, Alshare A, Shafirstein G, Bischof J (2018). A three-dimensional transient computational study of 532-nm laser thermal ablation in a geometrical model representing prostate tissue. Int. J. Hyperth..

[19] Sudo K, Shimojo Y, Nishimura T, Awazu K (2022). Three-dimensional transient simulation of CO$$_{2}$$ laser tissue vaporization and experimental evaluation with a hydrogel phantom. J. Innov. Opt. Health Sci..

[20] Ao H (2008). Thermal coagulation-induced changes of the optical properties of normal and adenomatous human colon tissues in vitro in the spectral range 400–1100 nm. Phys. Med. Biol..

[21] Jiang S, Zhang X (2005). Dynamic modeling of photothermal interactions for laser-induced interstitial thermotherapy: Parameter sensitivity analysis. Lasers Med. Sci..

[22] Bianchi L, Korganbayev S, Orrico A, De Landro M, Saccomandi P (2021). Quasi-distributed fiber optic sensor-based control system for interstitial laser ablation of tissue: Theoretical and experimental investigations. Biomed. Opt. Express.

[23] Tran VN, Truong VG, Lee YW, Kang HW (2020). Effect of optical energy modulation on the thermal response of biological tissue: Computational and experimental validations. Biomed. Opt. Express.

[24] Izumoto A (2020). Singlet oxygen model evaluation of interstitial photodynamic therapy with 5-aminolevulinic acid for malignant brain tumor. J. Biomed. Opt..

[25] Flock, S. T., Patterson, M. S., Wilson, B. C. & Wyman, D. R. Monte Carlo modeling of light propagation in highly scattering tissues. I: Model predictions and comparison with diffusion theory. *IEEE Trans. Biomed. Eng.* **36**, 1162–1168 (1989). 10.1109/tbme.1989.1173624 2606490

[26] Zhu C, Liu Q (2013). Review of Monte Carlo modeling of light transport in tissues. J. Biomed. Opt..

[27] Periyasamy V, Pramanik M (2017). Advances in Monte Carlo simulation for light propagation in tissue. IEEE Rev. Biomed. Eng..

[28] Iizuka MN, Vitkin IA, Kolios MC, Sherar MD (2000). The effects of dynamic optical properties during interstitial laser photocoagulation. Phys. Med. Biol..

[29] Pearce JA (2009). Relationship between Arrhenius models of thermal damage and the CEM 43 thermal dose. Proc. SPIE.

[30] Noguchi T, Hazama H, Nishimura T, Morita Y, Awazu K (2020). Enhancement of the safety and efficacy of colorectal endoscopic submucosal dissection using a CO$$_2$$ laser. Lasers Med. Sci..

[31] Nagarajan VK, Yu B (2016). Monitoring of tissue optical properties during thermal coagulation of ex vivo tissues. Lasers Surg. Med..

[32] Geoghegan R (2022). Interstitial optical monitoring of focal laser ablation. IEEE Trans. Biomed. Eng..

[33] He J (2019). A clinical prototype transrectal diffuse optical tomography (TRDOT) system for in vivo monitoring of photothermal therapy (PTT) of focal prostate cancer. IEEE Trans. Biomed. Eng..

[34] Cavagnaro M, Pinto R, Lopresto V (2015). Numerical models to evaluate the temperature increase induced by ex vivo microwave thermal ablation. Phys. Med. Biol..

[35] Jacques SL (1993). Role of tissue optics and pulse duration on tissue effects during high-power laser irradiation. Appl. Opt..

[36] Zhang S (2022). Modeling and ex vivo experimental validation of liver tissue carbonization with laser ablation. Comput. Methods Programs Biomed..

[37] Fasano A, Hömberg D, Naumov D (2010). On a mathematical model for laser-induced thermotherapy. Appl. Math. Model..

[38] Salomatina E, Yaroslavsky A (2008). Evaluation of the in vivo and ex vivo optical properties in a mouse ear model. Phys. Med. Biol..

[39] Wang KK-H, Zhu TC (2009). Reconstruction of in-vivo optical properties for human prostate using interstitial diffuse optical tomography. Opt. Express.

[40] Miyoshi Y, Nishimura T, Shimojo Y, Okayama K, Awazu K (2023). Endoscopic image-guided laser treatment system based on fiber bundle laser steering. Sci. Rep..

[41] Shaker HS, Shoeb MS, Yassin MM, Shaker SH (2012). Quartz head contact laser fiber: a novel fiber for laser ablation of the prostate using the 980 nm high power diode laser. J. Urol..

[42] Nguyen TH, Park S, Hlaing KK, Kang HW (2016). Temperature feedback-controlled photothermal treatment with diffusing applicator: Theoretical and experimental evaluations. Biomed. Opt. Express.

[43] Marti D, Aasbjerg RN, Andersen PE, Hansen AK (2018). MCmatlab: an open-source, user-friendly, MATLAB-integrated three-dimensional Monte Carlo light transport solver with heat diffusion and tissue damage. J. Biomed. Opt..

[44] Mohammadi A, Bianchi L, Asadi S, Saccomandi P (2021). Measurement of ex vivo liver, brain and pancreas thermal properties as function of temperature. Sensors.

[45] Fang Q, Boas DA (2009). Monte Carlo simulation of photon migration in 3D turbid media accelerated by graphics processing units. Opt. Express.

[46] Shimojo Y, Nishimura T, Hazama H, Ozawa T, Awazu K (2020). Measurement of absorption and reduced scattering coefficients in Asian human epidermis, dermis, and subcutaneous fat tissues in the 400- to 1100-nm wavelength range for optical penetration depth and energy deposition analysis. J. Biomed. Opt..

[47] Geoghegan R, Ter Haar G, Nightingale K, Marks L, Natarajan S (2022). Methods of monitoring thermal ablation of soft tissue tumors–a comprehensive review. Med. Phys..

[48] Tuchin, V. V. *Tissue Optics: Light Scattering Methods and Instruments for Medical Diagnosis* (SPIE, Bellingham, WA, USA, 2007), 2 edn.

[49] Shimojo Y, Nishimura T, Ozawa T, Tsuruta D, Awazu K (2023). Nonlinear absorption-based analysis of energy deposition in melanosomes for 532-nm short-pulsed laser skin treatment. Lasers Surg. Med..

