# Supplementary material for: Transient simulation of laser ablation based on Monte Carlo light transport with dynamic optical properties model
Source: Sci Rep. 2023 Jul 24;13:11898. doi: 10.1038/s41598-023-39026-4 (PMC10366136; doi:10.1038/s41598-023-39026-4)
Supplement: Supplementary file 3 — Supplementary Information 3. [file 41598_2023_39026_MOESM3_ESM.docx]

**Transient simulation of laser ablation based on Monte Carlo light transport with dynamic optical properties model**

Yu Shimojo^1,2,3^, Kazuma Sudo^2^, Takahiro Nishimura^2^, Toshiyuki Ozawa^1^, Daisuke Tsuruta^1^, and
Kunio Awazu^2,4^

^1^Graduate School of Medicine, Osaka Metropolitan University, Asahimachi 1-4-3, Abeno-ku, Osaka, 545-8585, Japan

^2^Graduate School of Engineering, Osaka University, Yamadaoka 2-1, Suita, Osaka, 565-0871, Japan

^3^Research Fellow of Japan Society for the Promotion of Science, Kojimachi 5-3-1, Chiyoda-ku, Tokyo, 102-0083, Japan

^4^Global Center for Medical Engineering and Informatics, Yamadaoka 2-2, Suita, Osaka, 565-0871, Japan

**Supplementary Information**

**Validation of the DOP model**

**Figure S1.** Root mean squared percentage error (RMSPE) between simulated and measured reduced scattering coefficients in the wavelength range of 600−100 nm.

**Vaporization and coagulation for the high-power irradiation settings**

**Movie S1.** Spatiotemporal changes in the simulated thermal damage distributions on the *zx* plane with *y* = 0 for irradiation power of 50 W and movement speed of 0.5 mm/s.

**Coagulation for the low-power irradiation settings**

**Movie S2.** Spatiotemporal changes in the simulated thermal damage distributions on the *zx* plane with *y* = 0 for irradiation power of 10 W and movement speed of 1.0 mm/s.

**Computational simulation of laser ablation**

**Figure S2.** Flowchart for computational simulation of laser ablation with the DOP model. *M*(**r**, *t*): numerical tissue model; *S*(**r**, *t*): light absorption distribution; *H*(**r**, *t*): enthalpy distribution; *T*(**r**, *t*): temperature distribution; Ω(**r**, *t*): damage parameter distribution; *∆t*: time step for thermal diffusion and thermal damage calculations; *i*_heat_: iteration count for thermal diffusion and thermal damage calculations; *j*_light_: iteration count for light transport calculations; *t*_heat_: calculation time for thermal diffusion and thermal damage with little change in Ω during light transport; *t*_laser,on_: calculation time during laser irradiation; *t*_laser,off_: calculation time after laser irradiation; and *t*_total_: total calculation time.

**Numerical model of tissue**

| Parameter | Air | Tissue | | Reference |
| --- | --- | --- | --- | --- |
|  |  | Native | Coagulated |  |
| Reduced scattering coefficient *μ*′_s_ (mm^−1^) | 0.01 | 0.51 | 1.85 | 1 |
| Absorption coefficient *μ*_a_ (mm^−1^) | 1.0×10^−5^ | 0.07 | | 1 |
| Anisotropy factor *g* (-) | 1.0 | 0.9 | | 2, 3 |
| Refractive index *n* (-) | 1.0 | 1.44 | | - |
| Thermal conductivity *k* (W/(cm·K)) | 2.602×10^−4^ | 5.43×10^−3^ | | 3 |
| Specific heat capacity *c*_p_ (J/(g·K)) | 1.006 | 3.542 | | 3 |
| Density *ρ* (g/cm^3^) | 1.196×10^−3^ | 1.0 | | 4 |
| Frequency factor *A* (1/s) | - | 7.39×10^37^ | | 5 |
| Activation energy *E*_a_ (J/mol) | - | 2.577×10^5^ | | 5 |
| Gas constant *R* (J/(mol·K)) | - | 8.314 | | 4 |
| Vaporization temperature *T*_v_ (°C) | - | 100 | | 6 |
| Latent heat of vaporization *L*_v_ (J/g) | - | 2.257×10^3^ | | 7 |

**Table S1.** Optical and thermal properties input into the numerical tissue model.

**Numerical calculation of tissue coagulation to determine the bathing conditions**

Coagulation of the tissue was simulated numerically to prepare samples with various damage parameters (Ω = 1, 2, 3, 4, 5) for optical properties measurement. Figure S3 shows the simulation setup used for bathing. Liver tissue samples with dimensions of 15 mm × 1.5 mm × 15 mm were placed in a hot water bath. The temperature rise in the tissue was then calculated using the following equation:

| $\begin{aligned} \rho c_{p}\frac{\partial T\left( \mathbf{r},t \right)}{\partial t}=k\nabla^{2}T\left( \mathbf{r},t \right), \end{aligned}$ | (1) |
| --- | --- |

where *ρ* (g/cm^3^) is the density, *c*_p_ (J/(g·K)) is the specific heat capacity, and *k* (W/(cm·K)) is the thermal conductivity. The values of *ρ*, *c*_p_, and *k* for water were set at 0.997 g/cm^3^, 4.18 J/(g·K), and 5.98×10^−3^ W/(cm·K), respectively. The initial temperature of the samples was 22 °C. Ω was calculated from the temperature distribution using the Arrhenius integral. The bathing temperatures were set at 60 and 70 °C to evaluate the temperature dependence of the change in the tissue optical properties. Tissue shrinkage during bathing was not considered because no significant changes in the thicknesses of the samples before and after bathing were observed during the experiments. Table S2 shows the resulting bathing conditions used to prepare samples with Ω = 1, 2, 3, 4, and 5. Figure S4 shows the Ω distributions of the liver tissue. The spatial distributions in the tissue samples were homogeneous. Using the bathing conditions obtained here, the liver tissue samples were coagulated.

**Figure S3.** Three-dimensional numerical model consisting of liver tissue and water used to perform thermal damage simulations to determine the bathing conditions for the experiments (the *yz* plane and the *xz* plane are shown here).

|  | Damage parameter Ω | | | | |
| --- | --- | --- | --- | --- | --- |
| Bathing temperature | 1 | 2 | 3 | 4 | 5 |
| 60 °C | 360 s | 776 s | 1196 s | 1615 s | - |
| 70 °C | 30 s | 54 s | 78 s | 103 s | 126 s |

**Table S2.** Bathing conditions for preparation of samples with various values of damage parameter Ω.

**Figure S4.** Damage parameter distributions in the tissue calculated via computational simulations under various bathing conditions. The bathing times were set at (**a**) 360 s, (**b**) 776 s, (**c**) 1196 s, and (**d**) 1615 s at 60 °C, and at (**e**) 30 s, (**f**) 54 s, (**g**) 78 s, (**h**) 103 s, and (**i**) 126 s at 70 °C.

**Laser irradiation experiment**

**Figure S5.** Experimental setup. (**a**) Laser irradiation setup. (**b**) Spatial distribution of the laser beam at the sample surface, measured using a CMOS camera. Scale bar: 1 mm. (**c**) Comparison of the beam profiles obtained from the simulations and the experiments. The beam profiles showed Gaussian distributions. The beam profile for the experiment was obtained from a plot of the yellow dotted line shown in (b).

**Determination of the coagulation region in the experiment**

A coagulation region in the cross-sectional images was cut out based on the difference in the gray values between the native and coagulated tissues. The color images were converted into grayscale images using ImageJ. Representative areas of each image were then selected to obtain the gray value ratio of the coagulated tissue (Ω = 1) to the native tissue (Ω = 0), as shown in Fig. S6(a). The average gray value of the native tissue was then measured for each image from four undamaged areas in the irradiated sample; these areas are illustrated in Fig. S6(b). The average gray value obtained was multiplied by the ratio of the coagulated tissue to the native tissue to obtain the threshold gray value for coagulation for each image. The coagulation region was identified as the region in which the gray values exceeded the threshold gray value.

**Figure S6.** Determination of the coagulation region. (**a**) Comparison of the native and coagulated porcine liver tissue. The coagulated tissue was prepared by bathing at 60 °C for 360 s. The gray values of the native and coagulated tissues were measured within each rectangular area to obtain the gray value ratio of the coagulated tissue to the native tissue. (**b**) Four undamaged areas in the irradiated samples (indicated by the black rectangles) were selected to obtain an average gray value for the native tissue. The threshold gray value for coagulation was determined by multiplying the ratio obtained by the averaged gray value. Scale of the ruler: 0.5 mm.

**Measurement of the coagulation and vaporization regions**

The vaporization depths, widths, and areas, and the coagulation depths, widths, and areas of the irradiated samples were measured from the cross-sectional images. The vaporization depth was defined as the maximum distance from the tissue surface to the boundary between the vaporized area and the coagulated tissue. The vaporization width was defined as the maximum width of the vaporized region. The vaporization area was defined as the area of the vaporized region. The coagulation depth was defined as the length of the coagulation region on the same axis as the vaporization depth. The coagulation width was defined as the width of the coagulated region at the deepest part of the vaporization region. When vaporization was not observed, the coagulation depth was defined as the maximum distance from the tissue surface to the boundary between the coagulated tissue and the normal tissue, and the coagulation width was defined as the maximum width of the coagulated region. The coagulation area was defined simply as the area of the coagulated region.

**References**

1. Jacques, S. L. Coupling 3D Monte Carlo light transport in optically heterogeneous tissues to photoacoustic signal generation. *Photoacoustics* **2**, 137–142 (2014).
2. Tuchin, V. V. *Tissue Optics: Light Scattering Methods and Instruments for Medical Diagnosis* (SPIE, Bellingham, WA, USA, 2007), 2 edn.
3. Mohammadi, A., Bianchi, L., Asadi, S. & Saccomandi, P. Measurement of *ex vivo* liver, brain and pancreas thermal properties as function of temperature. *Sensors* **21**, 4236 (2021).
4. Tran, V. N., Truong, V. G., Jeong, S. & Kang, H. W. Computational analysis of linear energy modulation for laser thermal coagulation. *Biomed. Opt. Express* **9**, 2575 (2018).
5. Iizuka, M. N., Vitkin, I. A., Kolios, M. C. & Sherar, M. D. The effects of dynamic optical properties during interstitial laser photocoagulation. *Phys. Med. Biol.* **45**, 1335 (2000).
6. Elkhalil, H., Alshare, A., Shafirstein, G. & Bischof, J. A three-dimensional transient computational study of 532-nm laser thermal ablation in a geometrical model representing prostate tissue. *Int. J. Hyperth.* **35**, 568–577 (2018).
7. Blauth, S., Hübner, F., Leithäuser, C., Siedow, N. & Vogl, T. J. Mathematical modeling of vaporization during laser-induced thermotherapy in liver tissue. *J. Math. Ind.* **10**, 1–16 (2020).
